# Musculoskeletal pain in South African wheelchair basketball players of different point classifications

**DOI:** 10.17159/2078-516X/2019/v31i1a6067

**Published:** 2019-01-01

**Authors:** I S M Mateus, J D Pillay

**Affiliations:** 1 Department of Chiropractic, Faculty of Health Sciences, Durban University of Technology, South Africa; 2 Department of Basic Medical Sciences, Faculty of Health Sciences, Durban University of Technology, South Africa

**Keywords:** physical activity, athletes with impairments, prevalence, injury prevention

## Abstract

**Background:**

During recent years, wheelchair basketball has gained worldwide popularity. Several studies have demonstrated a high prevalence of injuries amongst wheelchair basketball players. Few studies, however, have investigated the prevalence of musculoskeletal pain in the context of different point classifications – an integral part of wheelchair sport.

**Objective:**

The aim of this study was to determine the prevalence of musculoskeletal pain in wheelchair basketball players of different point classifications in South Africa and to provide information on patterns of pain distribution in relation to point classification.

**Methods:**

A questionnaire was completed by 48 wheelchair basketball players participating in the SuperSport League Games of South Africa with point classifications ranging from 1.0 to 4.5. The results were used to determine the patterns of musculoskeletal pain distribution in relation to the different point classifications.

**Results:**

Forty-three completed questionnaires were analysed. The prevalence of total musculoskeletal pain was 58% (n=25). Shoulder pain presented the highest overall prevalence, regardless of point classification (n=23; 92% since the start of players’ wheelchair basketball careers and n=19; 76% over the last 12 months). It was found that lower point classification (1.0–2.5) players commonly experienced arm pain since the start of their wheelchair basketball careers (**η**=0.358), as well as specifically over the last 12 months (**η**=0.319), unlike higher point classification (3.0–4.5) players.

**Discussion:**

The study contextualises the prevalence of musculoskeletal pain to overall point classification in wheelchair basketball. Such data are important in the formation of injury prevention strategies, as athletes with impairments are predisposed to different types of musculoskeletal pain based on point classification.

Physical activity through sport is well recognised as an effective means of obtaining both physical and emotional health, as well as social well-being.^[1]^ Sport and recreation in general have an even greater impact on individuals with impairments who, partly due to the nature of their impairment, may be predisposed to leading sedentary lifestyles. Apart from the physical health and fitness benefits of sport, participation in either recreation or competitive sport promotes improved self-esteem and provides a sense of inclusion, thereby empowering participating individuals with impairments.^[2]^

Participation in sporting activities for individuals with impairments, especially wheelchair users, has increased exponentially over the past few decades despite the challenges faced by these athletes.^[3]^ Developments in Para sport is evident in the increase in the number of participants. In 1948, 16 participants competed in the first Stoke Mandeville Games and number quickly grew to over 4000 athletes competing in the Paralympic Games in London in 2012 ^[4]^ and in Rio in 2016 respectively. ^[5]^ There are a variety of wheelchair sports such as wheelchair tennis, road racing, track racing, wheelchair rugby and wheelchair basketball.

Wheelchair basketball is a fast-paced, five players per side sport. High intensity propulsion and manoeuvring are key characteristics of the sport, together with quick passing, rebounding and reaching overhead for shooting.^[6,7]^ Prior to participation, players are classified according to a point score system used to rank a player’s functional ability to execute movements in basketball. Ranking is largely dependent on their level of disability. This classification scale ranks players using a 1.0 to 4.5point range (eight classifications at 0.5 point intervals). Lower (1.0–2.5) point players are typically players that are wheelchair bound due to a spinal cord injury, spina bifida or other birth conditions/defects resulting in paralysis or the inability to use their lower limbs. Higher (3.0–4.5) point players include players with amputations, mild poliomyelitis or birth defects resulting in fairly significant leg length inequalities. These players may not necessarily require the use of a wheelchair for activities of daily living, but are required to use a wheelchair during competitive play. The five players on court at one time must not exceed 14 points as a team.^[8]^

Recent studies have shown that athletes with impairments are more prone towards experiencing musculoskeletal pain (MSP) and injury than able-bodied athletes.^[9]^ However, a more recent study by Derman et al. ^[10]^ established that there is a higher incidence of upper limb injuries in Para athletes as opposed to able-bodied athletes. Furthermore, the different wheelchair sports and the type of impairment of athletes may predispose them to varied incidences and types of MSP and injury.^[9]^ Some examples include lateral epicondylitis in wheelchair racers due to repetitive and forceful elbow extension, pronation and wrist flexion. Another example is De Quervain’s tenosynovitis in athletes that participate in racquet and throwing sports.^[11]^ In Italy, Bernardi et al.^[12]^ conducted a study on muscle pain in athletes with locomotor disability and found that 51% of wheelchair athletes reported muscle pain and that 59% of them were wheelchair basketball players. Due to the constant use of the upper extremity for wheelchair propulsion, wheelchair basketball players have an increased risk of pain and overuse injuries in this extremity, particularly of the shoulder.^[13]^ This is further exacerbated by overhead activities (for example, shooting or rebounding after a failed shot), ball handling, wheelchair manoeuvring and player pursuit.^[13]^ In addition, the seated position of these athletes, especially for those with spinal cord injuries, involves the pelvis being tilted posteriorly, with a forward head posture and an increased thoracic kyphosis.^[14]^ This causes the shoulder girdle to be displaced anteriorly, placing an increased strain on the neck and upper back, with decreased scapula-thoracic function.^[14]^ Furthermore, insufficient strengthening programmes of the stabilising muscles of the scapula and rotator cuff muscles may cause muscle imbalances in these athletes.^[9,14]^

Current research on MSP and other injuries in wheelchair basketball athletes primarily focusses on shoulder pain.^[14]^ Other regions of pain, however, include the wrist, neck and lower back due to high intensity movements in wheelchair basketball.^[11]^ Furthermore, MSP can be further affected by the type of disability with which the player presents^[9]^ and for which the risk for other injuries amongst the different point classifications may vary. The aim of this study was therefore to determine the prevalence and patterns of MSP distribution in relation to point classification in a cohort of athletes participating in wheelchair basketball.

## Methods

### Participants

The study population was comprised of 48 wheelchair basketball players, participating in the SuperSport League Games of South Africa, with point classifications ranging from 1.0 to 4.5.

### Procedure

A questionnaire, adapted from a study by Curtis and Black^[14]^ on female wheelchair basketball players, was used as the data collection tool in this study. The questionnaire included demographic data and disability characteristics, wheelchair basketball and activity levels, as well as data pertaining to the prevalence of MSP and its impact on wheelchair basketball and/or activities of daily living. For the purpose of this study, MSP was defined as an instance or multiple instances of pain in the musculoskeletal system. No distinction was made between acute, subacute or chronic pain, but rather MSP shown since the start of these players’ wheelchair basketball careers, and specifically in the last 12 months.

The questionnaire was critically appraised through a focus group discussion comprising six Para sport participants and specialists in questionnaire design. It was subsequently sent to a pilot group for administration. The focus group and pilot study was employed as an additional step to ensure that the measurement instrument would obtain the necessary data required to satisfy the objectives of the study. From the pilot study, a Cronbach’s alpha was obtained to determine the consistency of scoring. The result of the reliability coefficient for the entire questionnaire (comprised of 83 questions) generated a score of 0.73. A coefficient of 0.70 or greater is considered as an acceptable score for a standardised instrument.^[15]^

Permission to administer the questionnaire to the league players was obtained from the Chief Executive Officer/Secretary General of Wheelchair Basketball South Africa and the relevant management staff. Ethical approval was granted by the Durban University of Technology Institutional Research Ethics Committee (Clearance number IREC 059/15).

A letter of information was provided to all potential participants at the 2015 SuperSport League Games. Inclusion criteria incorporated signed informed consent by those participants who were wheelchair basketball players that had competed in the 2015 SuperSport League Games, were classified on a scale from 1.0 to 4.5 (based on International Classification Guidelines), who were a minimum of eighteen years of age and South African citizens.

The researcher attended the SuperSport League Games where the questionnaires were explained and subsequently given to all the players present. These questionnaires were completed anonymously and placed in a sealed ballot box.

### Statistical analysis

Questionnaire data were analysed using the SPSS statistical package, version 23.0. Inferential techniques included the use of correlations in the form of cross tabulations and eta scores, as these are typically used to present comparisons when one variable is nominal and the other is a scale measure (number). In this study, the association between point classification (the nominal variable) and pain prevalence (the scale measure) was investigated. Eta scores of 0.10 and less were interpreted to be a small/smaller than the typical strength of a relationship, 0.24 is a medium/typical strength, 0.37 is a large/larger than typical strength, and 0.45 is interpreted as a much larger than the typical strength of a relationship.^[16]^

## Results

Of the 48 wheelchair basketball players participating in the SuperSport League Games of South Africa, 43 participants completed the questionnaire. Almost half of the participants (n=20; 59%) were wheelchair users and most of them (n=41, 95%) had played wheelchair basketball for more than five years. Of the 43 participants, 25 (58%) had experienced MSP in the last 12 months. Of the 25 participants who had experienced MSP, 68% (n=17) attributed it to instances related to wheelchair basketball either during training or during a match. Furthermore, 80% of these participants (n=20) reported that MSP affected at least one aspect of their lives negatively, such as wheelchair basketball participation and performance, as well as work performance. Further analysis of the prevalence of MSP showed the results were stratified based on the point classification of players. There were seven 1.0, seven 2.0 and seven 3.0 point players. The different point players were therefore evenly spread from 1.0 to 4.5 points.

Table 1 illustrates the association between point classification and the prevalence of MSP since playing wheelchair basketball, whilst Table 2 illustrates the same association only over the last 12 months. Arm pain was markedly prevalent in both instances but only in lower point players.

There is a clear indication in Table 1 that the majority of the 35 players who had experienced MSP, encountered shoulder pain (n=23; 92%) since playing wheelchair basketball. Furthermore, the distribution of players experiencing shoulder pain does not appear to be limited to a specific point classification category.

The eta score for arm pain (**η**=0.358) showed a larger than typical relationship (an association) between point classification and the prevalence of arm pain since playing wheelchair basketball. This relationship was seen in Table 1 under the frequency distribution, which clearly showed that arm pain was more evident in the lower point players (1.0–2.5 point players). Furthermore it was noted that lower point players had the highest prevalence of MSP experiencing 39 out of 61 (64%) recorded instances of MSP.

There were no significant relationships between point classification and the prevalence of neck, back and lower extremity pain since playing wheelchair basketball, as displayed in Table 1 under the frequency distribution.

It is evident from Table 2 that the majority of the 25 players that experienced MSP, experienced shoulder pain (n=19; 76%) regardless of the point classification within the last 12 months.

The eta score showed a greater than typical relationship but less than large relationship between point classification and the prevalence of arm pain in the last 12 months (**η**=0.319). In Table 2, this relationship is shown where arm pain was experienced by two 1.0 point players. Again, it was noted that lower point players had the highest prevalence of MSP, with lower point players experiencing 30 out of 47 instances of MSP (64%).

This table shows that there was no significant relationship between point classification and the prevalence of neck, back and lower extremity pain in the last 12 months.

## Discussion

There was an evident association between the prevalence of arm pain and point classification. Lower (1.0–2.5) point players experienced arm pain more frequently than higher (3.0–4.5) point players since the start of their wheelchair basketball careers. Furthermore, hand and wrist pain was experienced more frequently by lower (1.0–2.5) point players (Tables 1 and 2 respectively). There was no association evident between the prevalence of neck, back, shoulder and lower extremity pain and point classification.

### Shoulder pain

Shoulder pain occurred in 23 participants (54%) since the start of their wheelchair basketball careers and was evenly distributed between lower point players (n=11) and higher point players (n=12). This finding is similar to that of the study by Yildirim et al^[13]^ where shoulder pain was found to be prevalent in 60% of lower point players and 58% of higher point players (p>0.05). Lower point players, as opposed to higher point players, generally use wheelchairs for activities of daily living, in addition to during training and matches, and therefore have excessive strain imposed on their upper extremities, particularly the shoulders.^[13]^ Contrary to this, higher point players assume more ball handling roles due to greater control of their trunks and are therefore involved more in shooting from further distances, rebounding and passing.^[13]^

### Arm pain

Arm pain occurred in four (9.3%) participants and was experienced more frequently by lower point players than by higher point players. This could be due to the position that lower point players mostly rely on the use of wheelchairs for mobility, thereby causing the upper limbs to bear the weight and remain continuously in use for propulsion.

### Hand and wrist pain

It was noted that hand and wrist pain occurred more predominantly in lower point players (n=4) than in higher point players (n=1). Studies have shown that the symptoms of carpal tunnel syndrome occur in 52–100% of wheelchair users,^[17]^ and activities involving wrist extension, such as transferring, propulsion, shooting and rebounding, may aggravate these symptoms resulting in hand and wrist pain.^[6]^ For example, Goosey-Tolfrey et al.^[6]^ found that different point players demonstrated altered shooting strategies. This could be a potential cause of the hand and wrist pain experienced, as this type of pain occurred more frequently in lower point players than in higher point players.

In this study, 68% of players reported that the pain was due to an incident during the sport, which indicates a need for a modification of training regimes and/or positioning of the various body parts to reduce pain and injury. In order to implement the most effective preventative measures, further studies that investigate the exact cause of each player’s pain are necessary. Such studies may require the use of three-dimensional kinematic data, in addition to pain prevalence data related to point classification. To illustrate this, Goosey-Tolfrey et al.^[6]^ obtained three-dimensional kinematic data from 15 male wheelchair basketball players. These players were grouped into a lower point (2.0–2.5) group and a higher point (4.0–4.5) group. Although this study did not investigate the prevalence of pain, the study revealed that lower point players generated greater angular velocity of the wrist at release of the free throw, whilst higher point players generated greater shoulder flexion angular velocity at the release of the free throw. Their findings concluded that different point players rely on different kinematic strategies which could be the basis of the prevalence of pain amongst players of different point classifications.

### Pain in the lower extremity

Although not as frequent as MSP in the upper extremity, it must be noted that lower point players also experienced MSP in the lower extremity, as indicated in Table 1 and Table 2 respectively. This could be due to the high intensity and fast-paced nature of the sport, ^[6]^ which may result in falls.

It is important therefore for both coaches and practitioners working with sports teams to identify and address biomechanical errors or deficiencies among the athletes with different impairments to ensure a full recovery or prevent injury.^[11]^

### Strengths of the study

This study highlights the difference in MSP patterns among players of different point classifications, an integral part of wheelchair sport which has not been considered in most other studies on wheelchair sport. Furthermore, to these authors knowledge, this study is the first in the South African context to consider MSP related to point classification in wheelchair sport.

### Limitations and recommendations

The study sample was small as it focused on South African wheelchair basketball players who participated in the SuperSport League Games. Furthermore, recruitment at the event limited the sample to elite players only, consequently resulting in recruitment bias. Future studies should include a wider range of players at different levels of performance.

While this study has used MSP as a proxy for tissue damage, the authors realise that that this is not always the case. To be able to evaluate whether tissue damage or injury occurs in the presence of pain a methodology which includes the reporting of pain and a physical assessment should be considered.

Furthermore, future studies should include a more detailed profile of the MSP experienced by wheelchair basketball athletes, including more in-depth questions around the incidence, severity, aetiology and mechanism of injury and pain.

## Conclusion

This study highlights the prevalence of MSP in wheelchair basketball athletes. Due to the increasing growth of wheelchair basketball, further research is necessary to strengthen these findings on MSP and injury in the context of the different point classifications, with particular reference to preventative strategies and their evaluation. In so doing, structured guidelines for reducing or preventing MSP in this group of athletes can be formulated and subsequently evaluated.

## Figures and Tables

**Table 1:** The association between point classification and the prevalence of musculoskeletal pain since playing wheelchair basketball

Region	Point Classification (n=25)								Total	Eta Score (η)

	1.0	1.5	2.0	2.5	3.0	3.5	4.0	4.5		
**Arm**	3	0	0	1	0	0	0	0	4	0.358*
**Hand and wrist**	1	2	1	0	0	0	1	0	5	0.211
**Shoulder**	4	1	3	3	0	4	5	3	23	0.200
**Thigh**	0	0	2	0	0	0	0	0	2	0.128
**Foot and ankle**	0	0	2	0	0	0	0	0	2	0.128
**Hip**	0	0	1	0	0	0	0	0	1	0.090
**Leg**	0	0	1	0	0	0	0	0	1	0.090
**Elbow**	1	1	0	2	0	0	0	1	5	0.086
**Low back**	2	0	1	1	2	1	1	0	8	0.072
**Knee**	0	0	1	0	0	0	1	0	2	0.062
**Upper back**	1	0	1	0	1	0	1	0	4	0.048
**Neck**	0	1	1	1	0	0	0	1	4	0.014
**Total**	12	5	14	8	3	5	9	5	61	

Data are expressed as number of participants in each classification.

* indicates strong relationship (larger than typical relationship) based on eta score >0.24.

**Table 2:** The association between point classification and the prevalence of musculoskeletal pain in the last 12 months

Region	Point Classification (n=25)								Total	Eta Score (η)

	1.0	1.5	2.0	2.5	3.0	3.5	4.0	4.5		
**Arm**	2	0	0	0	0	0	0	0	2	0.319*
**Low back**	2	0	0	2	1	1	0	0	6	0.147
**Thigh**	0	0	2	0	0	0	0	0	2	0.128
**Foot and ankle**	0	0	2	0	0	0	0	0	2	0.128
**Hip**	0	0	1	0	0	0	0	0	1	0.090
**Leg**	0	0	1	0	0	0	0	0	1	0.090
**Hand and wrist**	0	0	2	1	1	0	0	0	4	0.083
**Neck**	0	1	0	0	0	0	0	1	2	0.062
**Elbow**	1	0	0	0	1	0	0	1	3	0.037
**Upper back**	1	0	0	0	0	0	1	0	2	0.033
**Shoulder**	4	1	3	2	0	4	4	1	19	0.008
**Knee**	0	0	2	0	0	0	1	0	3	0.002
**Total**	10	2	13	5	3	5	6	3	47	

Data are expressed as number of participants in each classification.

* indicates strong relationship (larger than typical relationship) based on eta score >0.24.
